# Uncommon Sorption Mechanism of Aromatic Compounds onto Poly(Vinyl Alcohol)/Chitosan/Maleic Anhydride-β-Cyclodextrin Hydrogels

**DOI:** 10.3390/polym12040877

**Published:** 2020-04-10

**Authors:** Cesar M. C. Filho, Pedro V. A. Bueno, Alan F. Y. Matsushita, Bruno H. Vilsinski, Adley F. Rubira, Edvani C. Muniz, Dina M. B. Murtinho, Artur J. M. Valente

**Affiliations:** 1 Department of Chemistry, CQC, University of Coimbra, 3004-535 Coimbra, Portugal; alanmatsushita@hotmail.com (A.F.Y.M.); vilsinski@yahoo.com.br (B.H.V.); dmurtinho@ci.uc.pt (D.M.B.M.); 2 BRinova Biochemistry Lda., R. Fernanda Seno, 6, 7005-485 Évora, Portugal; 3 Grupo de Materiais Poliméricos e Compósitos (GMPC)-Departamento de Química, Universidade Estadual de Maringá, UEM, Maringá 87020-900, Brazil; pedro.bueno@live.com (P.V.A.B.); afrubira@gmail.com (A.F.R.); curtimuniz@gmail.com (E.C.M.); 4 Post-graduate Program on Materials Science & Engineering, Federal University of Technology, Paraná (UTFPR-LD), Londrina 86036-370, Brazil; 5 Department of Chemistry, Federal University of Piauí, Teresina CEP 64049-550, Brazil

**Keywords:** chitosan, maleic anhydride, poly(vinyl alcohol), β-cyclodextrin, monoaromatic hydrocarbons, polycyclic aromatic hydrocarbons

## Abstract

Aromatic hydrocarbons are extensive environmental pollutants occurring in both water and air media, and their removal is a priority effort for a healthy environment. The use of adsorbents is among the several strategies used for the remediation of these compounds. In this paper, we aim the synthesis of an amphiphilic hydrogel with the potential for the simultaneous sorption of a set of monocyclic and polycyclic aromatic hydrocarbons associated with toxicity effects in humans. Thus, we start by the synthesis of a copolymer-based in chitosan and β-cyclodextrin previously functionalized with the maleic anhydride. The presence of β-cyclodextrin will confer the ability to interact with hydrophobic compounds. The resulting material is posteriorly incorporated in a cryogel of poly(vinyl alcohol) matrix. We aim to improve the amphiphilic ability of the hydrogel matrix. The obtained hydrogel was characterized by swelling water kinetics, thermogravimetric analysis, rheological measurements, and scanning electron microscopy. The sorption of aromatic hydrocarbons onto the gel is characterized by pseudo-first-order kinetics and Henry isotherm, suggesting a physisorption mechanism. The results show that the presence of maleic anhydride-β-cyclodextrin and chitosan into hydrogels leads to an increase in the removal efficiency of the aromatic compounds. Additionally, the capacity of this hydrogel for removing these pollutants from a fossil fuel sample has also been tested.

## 1. Introduction

Monoaromatic hydrocarbons (e.g., benzene, toluene, and xylene isomers (BTXs)) and polycyclic aromatic hydrocarbons (PAHs; e.g., pyrene, benzo(b)fluoranthene (B(b)F), and benzo(a)pyrene (B(a)P)) are the priority and persistent micropollutants, due to their hydrophobic nature and toxicity [1,2,3,4,5,6].

These compounds are distributed mainly in the environment (co-occurring in water, air, and soil), resulting from different causes, including industrial wastes, incomplete combustion, or pyrolysis of numerous organic materials, fossil fuel spills, etc. [7,8,9]. BTXs and PAHs include recalcitrant, mutagenic, and carcinogenic compounds and, consequently, remediation strategies need to be developed for their treatment [4,6,8,10,11].

There is an overall lack of cost-effective traditional methods for the removal of these organic contaminants from water [12]. Mainly activated carbon (suggested by the United States Environmental Protection Agency—US EPA) [13], advanced oxidation processes (AOPs) [14], semiconductor photocatalytic degradants (based on ZnO, for example) [15], mesoporous metaled nanoparticles, biological treatments, and zeolites [16,17,18] have been used in the decontamination of environments with an excess of BTXs, PAHs, and other organic pollutants. However, some of the related processes are limited by the inadequate removal of trace amounts of aromatic hydrocarbons and the higher cost of pre- and post-treatment [16]. Hence, cheaper removal techniques and environmentally friendly sorbents are needed to allow an easier and secure disposal [13]. The advantages of these materials include their abundance and low cost (subsequently, they have slight aggregate value, compared to synthetic adsorbents) [12,19,20].

For example, recently, we have proposed the use of a methyltrimethoxyslane-derived aerogel-like adsorbent (MTMS) for BTXs and PAHs removal [8]. The material advantages are its simplicity, non-toxic characteristic, and environmental abundancy [8]. A considerable pollutant removal was verified due to hydrocarbon–hydrocarbon interactions between hydrophobic MTMS and pollutants (mainly π–π stacking). Material presented simultaneous and substantial removal capacity of BTXs, and other petroleum-derived compounds of a gasoline sample [8].

Hydrogels based on natural polysaccharides also have been used on the removal of petroleum-based materials. For example, Tucanboylu et al. developed a hydrogel of starch crosslinked with epichlorohydrin and glutaraldehyde for PAHs removal. The proposed materials presented high PAHs sorption (up to 1.42 g per gram of sorbent), being of low cost and simple preparation [21].

Chitosan ((1,4)-linked amino-2-deoxy-βD-glucan) is a non-toxic, biodegradable, and biocompatible polysaccharide derived from chitin [22] that is found naturally in the crustaceous and shellfish exoskeleton [23,24]. On the other hand, poly(vinyl alcohol) (PVA) is also nontoxic and shows bioadhesive characteristics [25]. Furthermore, PVA gels also exhibit a high degree of swelling in water and rubbery and elastic nature [26,27].

β-Cyclodextrins (β-CD) is a cyclic oligosaccharide obtained from the enzymatic degradation of starch and composed of seven glucose units [28,29]. β-CD have hydrophilic external surface and hydrophobic internal cavity that provides the amphiphilic characteristic of these molecules [30,31]. 

Maleic anhydride is a known monomer for copolymers formation purposes [32]. This moiety provides copolymers with a wide variety of options for chemical modification, which generates materials that act as drug delivery systems, polymer blending compatibilizers, and other applications [32].

Our research group also has developed hydrogels based on natural polysaccharide for petroleum derivative pollutant compounds [33]. For example, the sorption of hydrophobic compounds onto a hydrogel composed by chitosan and pectin functionalized with β-cyclodextrin (β-CD) and poly(vinyl alcohol) (PVA) was evaluated [33]. The results highlighted the higher sorption capacity of β-CD-and PVA-Pec/CS gels in comparison to the unfunctionalized material [28]. β-CD-Pec/CS gels showed higher sorption capacity than the PVA-Pec/CS material. That was assigned to the formation of the host–guest supramolecular structure between β-CD, and aromatic moieties present in pollutants [33].

The use of CS/PVA blend gels, prepared by the freezing/thawed cyclic method [34] shows some advantages, including improved mechanical strength and non-toxicity [35]. However, there is no research in the literature of a material constituted of those two polymers associated with the functionalization through the incorporation of maleic anhydride-β-cyclodextrin monomer (MA-β-CD) [36].

Here we aim to obtain a physically crosslinked hydrogel with enhanced amphiphilic properties. For that, the β-cyclodextrin is initially functionalized with maleic acid, followed by the reaction of this compound, by coacervation, with chitosan. The obtained copolymer is then incorporated in a poly(vinyl alcohol) matrix allowing it to obtain a freeze–thawing physically crosslinked hydrogel [34]. The influence of chitosan and MA-β-CD amounts, and their impact on the swelling properties of the composites hydrogels in different formulations were evaluated. The efficiency of the hydrogels for sorption of both BTXs and selected PAHs (pyrene, B(a)F, and B(a)P) has been measured. Finally, the capacity of this hydrogel to BTXs and PAHs removal from a real gasoline sample has also been measured and discussed.

## 2. Materials and Methods

### 2.1. Materials

β-cyclodextrin (>98%), poly(vinyl alcohol) (*M_w_* ca. 13,000; 98.0–98.8 mol% hydrolysis), *N,N*-dimethyformamide (DMF; 99%), acetone (99.5%), methanol (HPLC grade), and chloroform (99%) were obtained from Sigma-Aldrich (Darmstadt, Germany). Maleic anhydride (MA; 99%) was supplied by Acros Organics (Geel, Belgium). Chitosan with acetylation degree (DA) of 15 mol% (average molecular weight 87 × 10^3^ g mol^−1^) was purchased from Golden-Shell Biochemical (Yuhuan, China). Acetonitrile was purchased from Fisher Scientific (Loughborough, UK).

BTXs-Benzene (ben; >99.7%) and xylene (xyl; mixture of isomers; >97%) were purchased from Merck KGaA (Darmstadt, Germany), toluene (tol; >99.8%) were obtained from Lab-Scan (Gliwice, Poland). PAHs—Pyrene (pyr) gas chromatography (GC) grade (>97%), benzo(b)fluoranthene (B(b)F) HPLC grade (>98%), and benzo(a)pyrene (B(a)P; >96%) were purchased from Sigma-Aldrich (Darmstadt, Germany). Deionized water was obtained using a Millipore© system (Merck KGaA, Darmstadt, Germany). All reagents were used without further purification.

### 2.2. Synthesis and Characterization of the Cyclodextrin Functional Monomer (MA-β-CD)

**Scheme 1:**
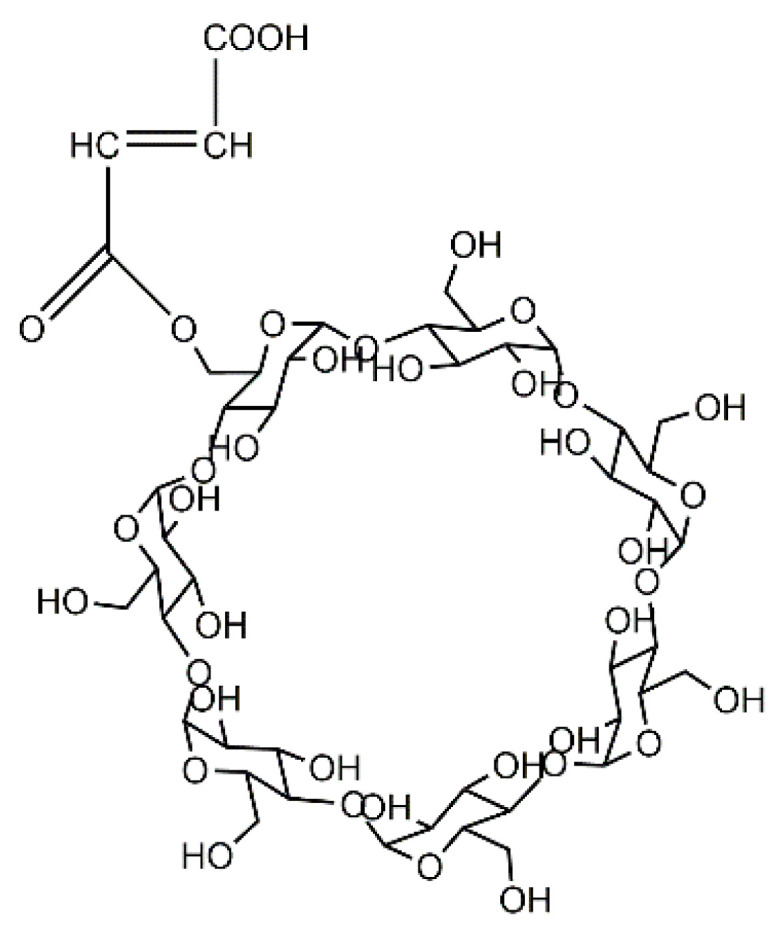
Molecular structure of MA-β-CD.

Maleic anhydride-β-cyclodextrin (MA-β-CD; Scheme 1) was synthesized following a previously described method [36,37]. Briefly, β-CD (5.71 g) and MA (4.90 g) were dissolved in 30 mL of dry DMF and stirred for 12 h at 80 °C in a round bottom flask, under a N_2_ atmosphere. The reaction was cooled to room temperature, and the product was precipitated with chloroform. The resulting beige solid (MA-β-CD) was obtained in 73% yield.

The proton nuclear magnetic resonance (^1^H NMR) spectrum of MA-β-CD was recorded on Bruker Avance III 400 NMR spectrometer (Billerica, MA, USA) by the dissolution of the samples in deuterium oxide (D_2_O, isotope substitution > 99.9% from Eurisotop, Cambridge, UK) and using ((3-(trimethylsilyl)-2,2′,3,3′-tetradeuteriopropionic acid), EurisoTop, as an internal reference.

#### Preparation of PVA/CS/MA-β-CD Hydrogels

PVA was added to ultrapure water and heated to 80 °C for 1 h to obtain a PVA solution (10 wt %). Chitosan (CS; 3 wt %) was dissolved in a suitable amount of acetate buffer (pH 4.5). MA-β-CD prepared in Section 2.2 was used without further preparation.

To evaluate the influence of parameters such as the concentration of CS, MA-β-CD, and PVA, as well as their effect on the swelling properties of hydrogels, surface response methodology (RSM) for two factors (feed concentrations of MA-β-CD and CS) was performed (see Table S2).

According to the designed blending ratio, the mixed solutions of CS, MA-β-CD, and PVA were stirred for 60 min—the time needed for obtaining a homogeneous solution. Then, 1 mL of the mixed solution was placed in wells of cell culture plates (see Figure S1A) and kept for 16 h at −20 °C to freeze, followed by thawing for 8 h at room temperature. This freeze–thawing cycle was repeated twice. The obtained hydrogel was dried by lyophilization for 24 h on a Free Zone 4.5 Liter Benchtop vacuum freeze-drying system (Kansas City, MO, USA) equipment (see Figure S1B).

### 2.3. Characterization of the Hydrogels

#### 2.3.1. Swelling Equilibrium Studies

The contribution of different factors (concentration of chitosan, PVA, and MA-β-CD) to the swelling degree (*Q*) of hydrogels was evaluated at 25 °C, in ultrapure water [38]. All experiments were carried out in triplicate.

The swelling of the hydrogels studies (equilibrium and kinetics) in water were studied by measuring the mass of swollen hydrogel and xerogel (*m_t_* and *m*_x_, respectively), at different times, *t*, by using Equation (1) [39] and following the procedure described in previous work [40].

(1) $$Q_{t}=\frac{\left(m_{t}-m_{x}\right)}{m_{x}}$$

#### 2.3.2. Thermogravimetric Analysis

Thermograms of hydrogels were obtained in a TG 209 F3 Tarsus thermogravimetric analyzer (Netzsch Instruments, Selb, Germany). Samples (ca. 10 mg) were weighed in alumina pans and were heated from 30 to 900 °C at a heating rate of 10 °C min^−1^ under N_2_ atmosphere (flow rate of 20 mL min^−1^).

#### 2.3.3. Surface Morphology

Surface morphologies of hydrogel samples were observed using a scanning electron microscope (Tescan-Vegas 3 SEM, Brno, Czech Republic). For that, samples were previously frozen in liquid N_2_, lyophilized, and sputter-coated with a thin gold layer. ImageJ^®^ software (National Institutes of Health, Bethesda, MD, USA) was used to measure the pore size.

#### 2.3.4. Rheological Experiments

Rheological measurements were performed using a Thermo Scientific HaakeMars III rheometer (Waltham, MA, USA), with an automatic gap setting and with a cone and plate geometry (35 mm, 1°). The temperature control (±0.1 °C) was achieved using a Peltier unit. The rheological properties of hydrogels were determined through oscillatory measurements. An amplitude sweep (1–200 Pa) at a fixed frequency (1 Hz) was first performed to make sure that the selected stress (20 Pa) is within the linear viscoelastic region. Then an oscillatory frequency sweep (0.1–10 s^−1^), at 25 °C, was performed to measure the storage (*G’*) and loss (*G”*) moduli, respectively. The *G’* and *G”* were also analyzed as a function of temperature (20–60 °C).

### 2.4. Sorption Studies

A stock solution of either BTXs or PAHs was prepared at a concentration of 100 mgL^−1^ by dissolving calculated amounts of individual analyte in methanol and stored in an amber glass at −20 °C. The stock solution was then diluted in ultrapure water: methanol (70:30 *v/v*) solution to obtain working solutions of appropriate concentrations (for more details, see Section 2.5). The equilibrium sorption was reached after 400 min. To be sure about this time, preliminary experiments were carried out during 1400 min (ca. 1 day). All experiments were carried out in triplicate.

#### 2.4.1. Sorption Isotherms

For sorption isotherms, 40 mL of BTXs and PAHs mixed solutions (see Table S1) were kept in contact with a given amount (ca. 100 mg) of hydrogels based on PVA/CS/MA-β-CD. The flasks were covered with aluminum sheets to avoid the oxidation and photodegradation of PAHs [41] and were kept firmly closed in order to prevent the hydrocarbon volatilization. To refrain the dispersion of the hydrogel, a Nylon tea-bag (100-mesh Nylon screen) was used [42,43]. The solid–liquid systems were kept at 25 °C (using a VelpScientifica thermostatic bath, Vantaa, Finland) under continuously stirring (400 rpm) during ca. 20 h in order to guarantee that the sorption equilibrium is attained.

For comparative purposes, hydrogels that showed lowest, intermediate, and highest swelling degrees were used.

The adsorption capacity (*q*_e_), expressed as adsorbate removal per unit mass of adsorbent (mg g^−1^), is calculated according to Equation (2).

(2) $$q_{e}=\frac{V\left(C_{0}-C_{e}\right)}{m_{x}}$$

where *V* is the volume of the solution, and *C_0_* and *C_e_* are the initial and equilibrium concentrations (in mg mL^−1^) of the analytes (BTXs and PHAs) in mixed solutions, respectively. 

The removal efficiency (*RE*) was calculated according to the following equation:

(3) $$RE\left(\%\right)=\left(\frac{C_{o}-C_{e}}{C_{0}}\right)\times 100$$

To further evaluate the individual influence of PVA, CS, and MA-β-CD on the simultaneous removal efficiency (RE) of BTXs and PAHs, PVA hydrogels (10 wt %) and hydrogels PVA/CS and PVA/MA-β-CD were prepared (CS and MA-β-CD containing corresponding to the amounts found in the PVA/CS/MA-β-CD composite gels (with the different swelling degrees: low (LS), intermediary (IS) and high (HS)). See Table S1 to the concentrations of the aromatic hydrocarbons used in the sorption with these reference hydrogels (blanks).

#### 2.4.2. Sorption Kinetics

The sorption kinetics were performed at 25 °C by using a thermostatic bath (VelpScientifica) under continuously stirring (400 rpm), during ca. 19 h. The blends were kept in contact with the composite hydrogels based on PVA/CS/MA-β-CD (IS; ca. 100 mg). The effect of concentrations of hydrocarbons (BTXs and PAHs) on the sorption was studied (see Table 1). Other experimental details are similar to those described in the previous section.

The capacity of hydrogels for the removal of BTXs and PAHs existing in a real gasoline sample has also been evaluated. For that, composite hydrogels (IS (ca. 10 mg) supported by a Nylon tea-bag were introduced in a screw-caps glass tube. Afterward, a MeOH:H_2_O mixture (70:30 *v/v*), commercial gasoline diluted in methanol (ca. 1500 times) and BTXs and PAHs standard solutions with average concentrations of 3 mg L^−1^ and 1 mg L^−1^, respectively, were added, with a final volume of 40 mL. The mixture was stirred at 25 °C for one day. The amount of the BTXs and PAHs adsorbed onto blend hydrogels, in each run, was determined by measuring the concentration of adsorbed BTXs and PAHs (mg L^−1^) in the supernatant solution, before and after sorption.

#### 2.4.3. Reusability of the Composite Hydrogels

For the desorption studies, hydrogels based on PVA/CS/MA-β-CD samples (IS), previously loaded with BTXs and PAHs, were collected and transferred to glass tubes with 40 mL of a MeOH:H_2_O (70:30 *v/v*) mixture, and acidified to pH 3 (using HCl 1 M), and left stirring (at 450 rpm) for 300 h, at 25 °C (labeled as Des. 1). The concentrations of BTXs and PAHs were then measured by HPLC, and the desorption ratio (*DR*) was calculated according to the following equation:

(4) $$DR\left(\%\right)=\left(\frac{m_{r}}{m_{0}}\right)\times 100$$

where *m*_r_ and *m*_0_ are the amounts of BTXs and PAHs desorbed and sorbed, respectively.

The hydrogels used in the desorption process (Des. 1) were placed again in contact with the BTXs and PHAs mixed solutions in MeOH:H_2_O (70:30 *v*/*v*), as described above. The 2nd desorption cycle (Des. 2) was over after 4 days, and the concentrations of the hydrocarbons were measured as previously described.

### 2.5. Analytical Procedure

In all sorption–desorption cycles, the analysis of BTXs and PAHs was done in a VWR-Hitachi LaChrom Elite HPLC system (Tokyo, Japan), equipped with a diode array detector (DAD), autosampler, degasser and column oven [44].

The best results were obtained by using an analytical column (0.25 m × 4.6 mm, 5 µm film) Purospher^®^ Star RP-18 endcapped (Merck-Millipore, Darmstadt, Germany). Calibration curves were daily prepared.

## 3. Results and Discussion

### 3.1. Synthesis of Maleic Acid-β-Cyclodextrin (MA-β-CD)

**Figure 1:**
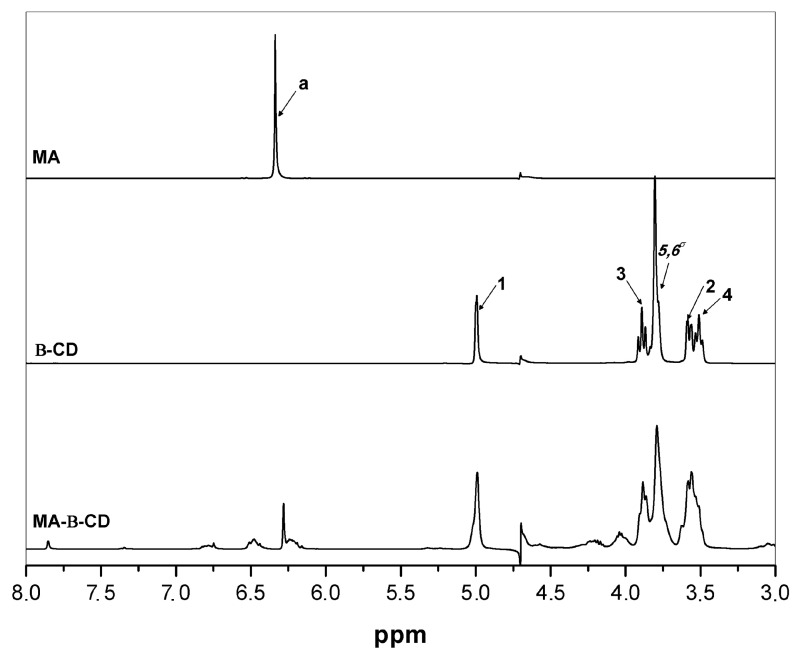
^1^H NMR spectra of MA, β-CD and MA-β-CD (from top to bottom) in D_2_O, 400 MHz.

The functionalization of β-cyclodextrin with maleic acid was characterized by ^1^H NMR spectroscopy, by comparison of β-CD, MA, and MA-β-CD spectra (see Figure 1). The ^1^H NMR spectrum of β-CD shows resonances occurring at δ 3.77–3.88 ppm, assigned to H_3_ atoms located at the wide side of the cavity of the β-CD. Additionally, a doublet at δ 4.99 ppm, assigned to H_1_ atoms (located outside the cavity between H_4_ and H_2_ atoms) [37] is shown as well. The overlapping resonances for the H_5_, H_6′_, and H_6′’_ atoms are found at δ 3.66–3.76 ppm [37,45]. In the ^1^H NMR spectra, the protons of maleic anhydride can be found at δ 6.34 ppm [46]. Resonance at δ 6.28 ppm can be visualized in ^1^H NMR spectrum of MA-β-CD and assigned to MA. Resonances at δ 4.99 ppm and δ 3.86–3.55 ppm were also observed in such spectrum and were due to β-CD. These results proved that the expected chemical modification due to the insertion of MA effectively occurred in the structure of β-CD.

By using ^1^H NMR spectra, and considering the equation reported elsewhere [47], the functionalization yield was computed and was equal to 28% (mol/mol) of MA.

FTIR analysis was also carried out to determine the interactions between MA and β-CD. Figure S2 shows the FTIR spectra of β-CD (I), MA (II), and MA-β-CD (III). The β-CD spectra was characterized by the following vibrational bands: (i) the broad band observed at 3408 cm^−1^ and the band at 2928 cm^−1^ that were assigned to the O-H stretching vibration and to the C-H stretch of alkyl groups of β-CD, respectively; and (ii) the bands observed at 1648 cm^−1^ and 1020 cm^−1^ that were assigned to the asymmetric stretching and stretching, respectively, of C-O [48,49,50].

The FTIR spectrum of MA shows typical bands at 1586 cm^−1^ (C = C stretching), 1772 cm^−1^ and a weak band 1856 cm^−1^ (symmetric and asymmetric C = O stretching respectively), and 3190 cm^−1^ (C-H stretching vibrations) [48,51].

Some typical bands of CD (1064, 1659, and 2940 cm^−1)^ remained unaffected in the IR spectrum of MA-β-CD, suggesting that the structure of β-CD remains almost unchanged upon functionalization [52]. In addition, the peaks at 1659 and 1737 cm^−1^, due to MA, may be the main evidence of the incorporation of MA in the β-CD backbone [33,53,54,55,56]. Both ^1^H NMR and FTIR spectroscopies revealed β-CD modification with the insertion of MA. 

### 3.2. Experimental Planning and Statistical Analysis

In order to perform a systematic study of the influence of the independent variables on the swelling of hydrogels (see Section 3.3.1), experimental and predicted values were compared with empirical equations adjusted with the help of analysis of variance (ANOVA), at the 95% confidence level (*p* < 0.05), by using the software Design Expert^TM^ (trial version). For this, the criterion of hierarchy was taken into account and the equation obtained to predict the equilibrium swelling values (*Q*_eq,exp_; Equation (5)) is written as a function of the coded terms of the independent variables:

*Q*_eq_ = 4.64 + 4.23*A* + 3.01*B* + 10.33*AB* (5)

where *A* and *B* are the concentrations of CS and MA-β-CD, respectively, presented as coded terms of the independent variables.

Table S3 shows the results of the analysis of variance for the response predicted by Equation (5), revealing that the adjusted model was not significant according to the *F* test (*p* > 0.05). Additionally, the *F*-value of 5.68 also implies that the model was not significant. This indicates a more complex dependency of each variable in the swelling behavior.

**Figure 2:**
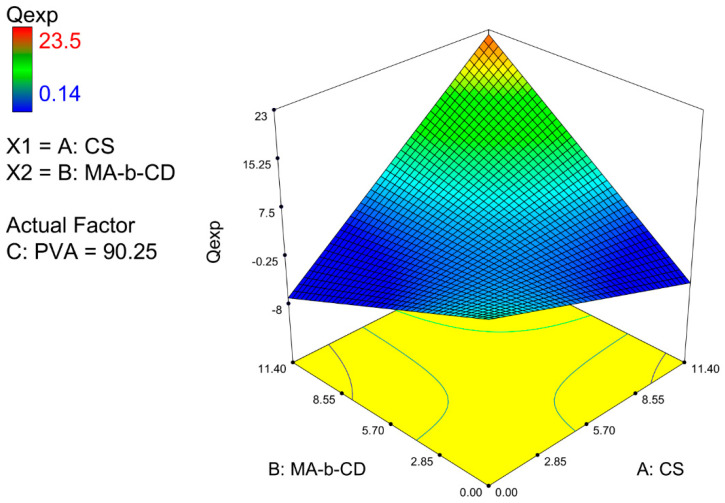
Three-dimensional response surface for the swelling ratio at equilibrium versus concentration of CS and MA-β-CD.

The response surface profile (see Figure 2) shows a region of maximum predicted values around 23.5 for *Q_e_* value. In fact, 55% of the variability of the response can be explained by the model. This type of graph is widely used to simultaneously represent the effects of variables along with a dependent variable (response), and thus an integral analysis of the influence of interactions between all independent variables on both responses can then be better visualized. The strong interaction effect can be visualized on a highly inclined surface for achieving the maximum swelling values.

### 3.3. Characterization of Hydrogels

#### 3.3.1. Swelling Degree

**Figure 3:**
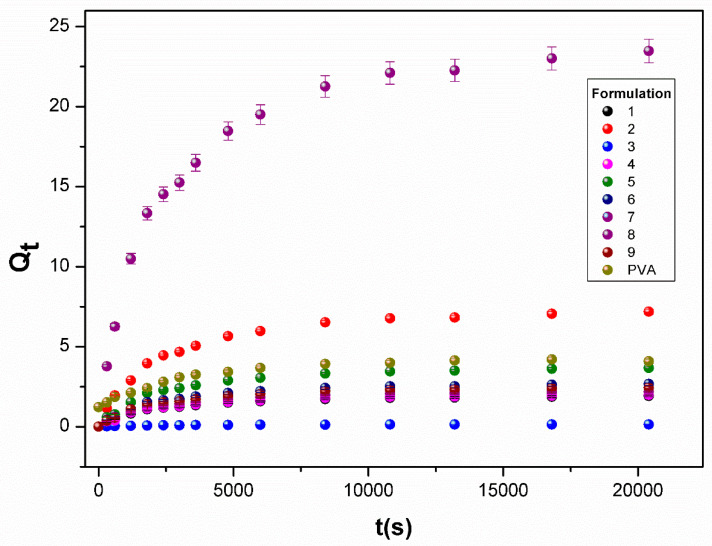
Swelling kinetics of PVA/CS/MA-β-CD composite hydrogels (see Table S2 in water, at 25 °C).

The effect of CS and MA-β-CD on the structure of the hydrogel has been further assessed through the water swelling degree quantification (see Figure 3). It can be seen from the analysis of *Q*_e_,_exp_ values (see Table 2) that all components could significantly affect the swelling capacity of the hydrogels. However, the highest *Q*_e, exp_ value was observed for the formulation 8 (Table 2), while the hydrogel obtained by using the formulation 3 had the lowest swelling degree. It is necessary to mention that hydrogel of formulation 8 had a slightly higher swelling degree (*Q*_e,exp_ = 23.5) in comparison to the pectin-PVA/CS hydrogel (*Q*_e,exp_ = 23.0) reported elsewhere [33]. These results show that CS and also MA-β-CD are essential to affect the swelling behavior of hydrogels, in agreement with the analysis done in Section 3.2.

It should be probable that the amphiphilic compounds: β-CD and PVA will contribute to the occurrence of a more porous and heterogeneous blend highly dependent on the gel formulation [27,33,57].

In order to get an insight on the water sorption mechanism, the swelling kinetics were evaluated using first- and second-order kinetics equations [58] (Equations (6) and (7), respectively):

(6) $$ln\left(\frac{Q_{e}}{Q_{e}-Q_{t}}\right)=k_{1}t$$

(7) $$ln\left(\frac{Q_{e}}{Q_{e}-Q_{t}}\right)=k_{1}t$$

where *k*_1_ and *k*_2_ are the swelling rate constants, and *Q*_t_ and *Q*_e_ are the swelling ratios at time *t* and equilibrium conditions, respectively. The fitting values are shown in Table 2. The best model was chosen through the Akaike’s information criteria (*AIC*—Equation (8)) [59]:

(8) $$AIC=nlog\left(\frac{s^{2}}{n}\right)+2K$$

where *s*^2^ is the residual sum of squares, *n* is the number of experimental data points, and *K* is the number of model parameters.

The analysis of the data summarized in Table 2 shows that, for all formulations, the swelling kinetics follows a pseudo-first-order kinetic mechanism, suggesting that water–water interactions are more prominent than water-polymer interactions [58].

From the analysis of Figure 2, it comes out that PVA and chitosan act on opposite directions on the stiffness and *Q*_e_, i.e., by increasing the amount of CS in the hydrogel both *Q*_e_ and stiffness increase (Figure 2). On the other hand, by increasing the amount of PVA, both *Q*_e_ and stiffness decrease. This is in close agreement with rheological analysis (see Section 3.3.4), and the hypothesis is that the polymeric crosslinkers with variable chain flexibility contribute to a close dependence of the elastic modulus on the swelling degree of hydrogels [60].

#### 3.3.2. Thermogravimetric Analysis

**Figure 4:**
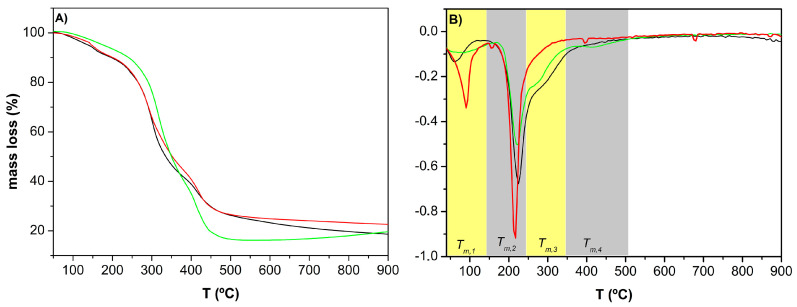
Thermograms (**A**) and derivative thermogravimetry (DTG; (**B**) of composite hydrogels based on PVA/CS/MA-β-CD, which presented a low (^___^), intermediate (^___^), and high (^___^) swelling degree, respectively.

The effect of hydrogel composition and swelling degrees (LS, IS, and HS) for MA-β-CD, CS and PVA, respectively, on the thermal stability of hydrogels was evaluated by TGA (Figure 4A). By comparing the *T_m,i_* (the maximum degradation rate at temperature range *i*) for different gels at a giving temperature range *i*, it can be concluded that the monomer concentration has an important effect on the thermal performance of blends. The first degradation phase (*T*_m,1_): 63, 93, and 87 °C, for LS, IS, and HS, respectively, might be due to the loss of adsorbed water in the samples. Concerning the main degradation step (*T*_m,2_), it has been found that for IS: *T*_m,2_ = 216 °C. This is accompanied by the incidence of shoulders in the DTG curves (Figure 4B at temperatures around 270 °C (*T*_m,3_)), which might be related with the chitosan degradation temperature [61]. Further, the functionalized MA-β-CD have the weight loss at 300 °C, consistent with the weight loss of β-CD [62]. 

#### 3.3.3. Surface Morphology Studies

**Figure 5:**
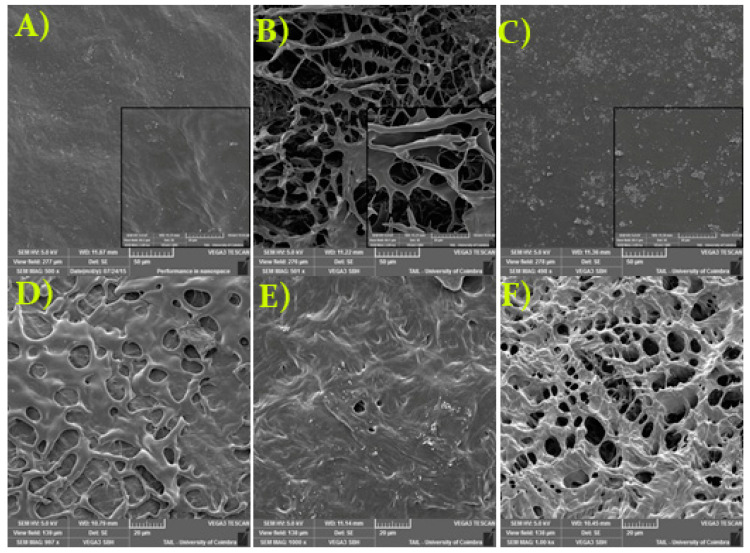
Scanning electron micrographs of composite hydrogels based on PVA/CS/MA-β-CD at 500× magnification, which presented a low (**A**), intermediate (**B**), and high (**C**) swelling degree. Insert figures, SEM images at 2000× magnification. Blanks-PVA (100%; (**D**); PVA (88.6% *v*/*v*) + CS (11.4% *v*/*v*; (**E**); and PVA (94.25% *v*/*v*) + MA-β-CD (5.75% *w*/*v*; (**F**), at 1000× magnification.

The surface morphology of the freeze-dried PVA/CS/MA-β-CD composite hydrogels with different swelling degrees was also studied by SEM (Figure 5). Though, important differences in the surface morphology occur according to the degree of swelling. The *IS* hydrogel surface (Figure 5B) shows a porous network structure with pore size range from 5.7 to 36.1 µm. The presence of this porous surface in the composite gel may have contributed to the higher sorption of the aromatic hydrocarbons (see Section 3.4.1). The observation of the surface morphology of reference hydrogels (blanks; Figure 5D–F), shows a porous hydrogel, with a pore size range from 3.5 to 34.4 µm (PVA—Figure 5D), 4.4–13.2 µm (PVA + CS—Figure 5E), and 4.1–44.6 µm (PVA + MA-β-CD—Figure 5F); therefore, it is worth noting to conclude that the presence of MA-β-CD was directly related to the increase in the size and number of pores on the hydrogels surface.

#### 3.3.4. Rheological Properties

**Figure 6:**
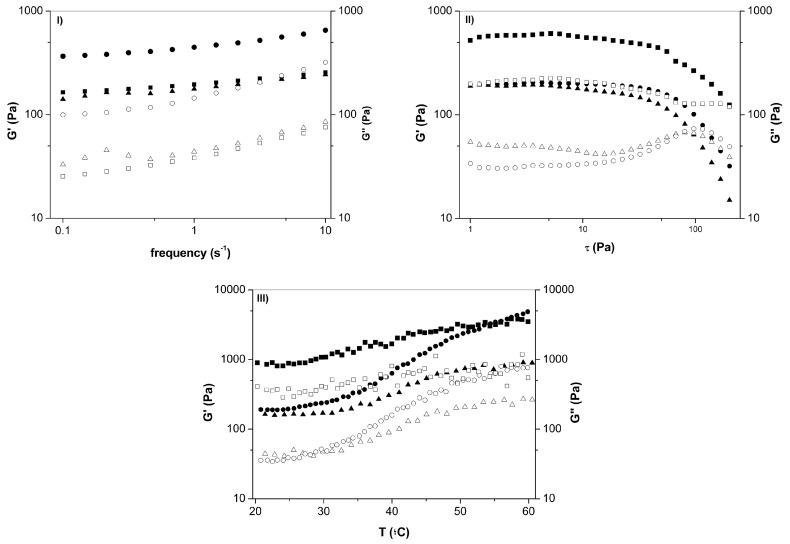
Rheological properties of PVA/CS/MA-β-CD composite hydrogels, which presented high (●,o), intermediate (▪,≤), and low(▲,ρ) swelling degree, respectively, determined through oscillatory measurements, oscillatory frequency sweep (0.1–10 Hz; **I**), amplitude sweep(1–200 Pa; **II**), and as a function of temperature (20–60 °C; **III**).

In order to evaluate the influence of hydrogel composition on the viscoelastic properties of hydrogels, rheological experiments were carried out and the results are shown in the Figure 6. Firstly, oscillatory measurements were carried out at 25 °C; the frequency sweep (τ = 1 Pa) showed a typical viscoelastic behavior at the whole range of applied frequency. It was verified that the gels exhibited a typical solid-like rheological behavior, being the values of the storage modulus *G’* higher than the loss modulus *G´´* [63,64], as expected. Amplitude sweep measurements (*f* = 1 Hz) followed a similar trend, with a weak dependence of the *G´* and *G´´* in most of the applied stress range. At the higher stress values (τ >150 Pa), the loss modulus increased until the intersection with the storage modulus, indicating a phase transition phenomenon. There is a difference in the stiffness of the hydrogels, and the mechanical strength is higher for the hydrogel with intermediate swelling degree. It can be concluded that the greater the amount of CS in the greater is the gel’s stiffness. Moreover, Figure 6 also shows the thermoresponsive behavior of hydrogels, i.e., upon heating, *G´* and *G´´* remained nearly constant, with *G´* greater than *G´´*, which indicate that the hydrogels are very stable in this temperature range (20–60 °C).

### 3.4. Sorption Studies

#### 3.4.1. Sorption Kinetics

**Figure 7:**
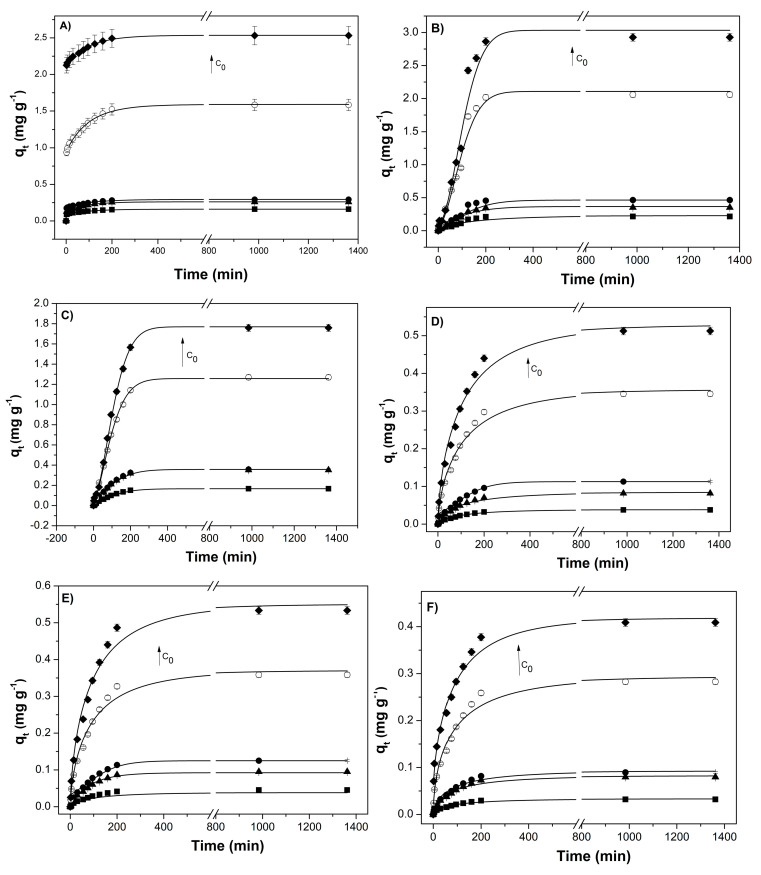
Sorption kinetic of mono-and polycyclic aromatic hydrocarbons, in mixed solutions (benzene (**A**), toluene (**B**), xylene (**C**), pyrene (**D**), B(b)F (**E**), and B(a)P (**F**)), at 25 °C, onto the PVA/CS/MA-β-CD hydrogel (intermediate swelling degree), and for several hydrocarbons *C*_0_ values (the arrow indicates the direction of higher concentrations).

Sorption kinetics studies were performed to obtain the time necessary for the adsorption process and to evaluate the limiting step on the adsorption mechanism. Figure S3 presents the sorption kinetic of BTXs and some PAHs present in multicomponent mixtures, at different initial concentrations, by the PVA/CS/MA-β-CD hydrogel with an intermediate swelling degree (Figure 7).

Sorption kinetic profiles were similar for different aromatic compounds (and different initial concentrations), with the equilibrium time being attained at ca. 400 min. This relatively fast adsorption is relevant, especially for the treatment of running wastewater [53]. It is worth noticing that our equilibrium time is lower than that observed for other polysaccharide-based-hydrogels. For example, Tucanboylu et al. showed that hydrogels based on starch reached the equilibrium-time in PAHS adsorption only after 3 days [21].

Benzene, toluene, and xylene show lower sorption amounts at higher initial concentrations. This suggests that the adsorption capacity can be affected by the interaction degree between adsorbate–adsorbent and mainly by adsorbate–absorbate interactions [53]. The PHAs show smaller interaction with the hydrogel than the BTXs [63]. Besides, the interaction between aromatics is likely more significant than the adsorbate–adsorbent one [65]. Once the knowledge of the initial and border conditions, in the kinetics experiments, are not very well defined, due to the complexity of the system, the sorption kinetics will be evaluated by the pseudo 1^st^ order and pseudo 2nd order kinetic models [66,67] (Equations (6) and (7), by substituting *Q_e_* and *Q_t_* by *q_e_* and *q_t_* (mg g^−1^), respectively).

The fitting parameters of Equations (6) and (7) to experimental sorption for BTXs and PAHs, and the corresponding AICs, are summarized in the Figure S3 and Table S4. The results revealed that the sorption kinetics for all adsorbates followed a pseudo-first order model. In addition, the sorption capacities in equilibrium (*q_e_*) obtained using the kinetic models match well with the experimental data. This suggests that physisorption is the limiting step of the sorption process and is characterized by semireversible sorption/desorption cycles [8,68]. This model further indicates that the binding of adsorbate to the surface of the adsorbent involves a relatively weak interaction that can be attributed to van der Waals forces or hydrophobic interactions, which are similar to the forces of molecular cohesion [69]. This suggests that PVA play a major role on the adsorption process. Molecular dynamic simulations on PVA and simvastatin (a slightly hydrophobic drug) systems show that PVA molecules assume a configuration where the hydroxyl groups are positioned away from the drug, allowing the occurrence of hydrophobic interactions between the drug and the PVA backbone [27]. A similar mechanism can also occur between the aromatic hydrocarbons and the PVA. It should be noted that hydrophobic interactions between the adsorbates and cyclodextrins is also expected. Besides, we cannot neglect that PAHs contain aromatic rings with π-acceptor and π-donor properties that are charged at environmental pH. Thus PAHs compounds are electron-rich and interact strongly with π-accepting sites of adsorbents that are electron-deficient [70]. Here, nitrogen groups present in chitosan (from units of 2-amino-2-deoxy-D-glucopyranose and residual 2-acetamido-2-deoxy-D-glucopyranose) can act as π-accepting sites for PAHs and BTXs compounds [70]. Furthermore, π–π interactions between adsorbates can also occur.

#### 3.4.2. Sorption Isotherms

In order to go deeper on the sorption mechanism, Freundlich [71,72] and Henry [73] models have been fitted to the experimental sorption data for the hydrogel with intermediate swelling degree. These models can be expressed by using the Equations (9) and (10), respectively.

(9) $$q_{e}=K_{F}C_{e}{}^{1{/}_{n_{F}}}$$

(10) $$q_{e}=K_{H}C_{e}$$

where *K*_F_ and *K*_H_ are the Freundlich and Henry constants and *n*_F_ is a constant related with surface heterogeneity [72].

Figure S4 shows the sorption isotherms fitted by the two models for the six aromatic compounds mixtures. Table 3 shows the fitting parameters as well as both the determination coefficients and the *AIC* for the fitting of Equations (9) and (10) to the experimental data. It can be concluded that Equation (10) shows better fitting to the experimental data. This is in agreement with the previous section, showing that kinetic adsorption is governed by physisorption. This also shows that the interaction between the solute–polymer and solute–solute is weak when compared to the polymer–polymer interaction [74], which corresponds to a condition in which the adsorbed phase shows a degree of dilution with no interactions among the adsorbate molecules, as well as competition for the adsorptive sites. Additionally, the Henry constant (K_H_) provides important information about the partition of adsorbate between the gel and aqueous phases.

**Figure 8:**
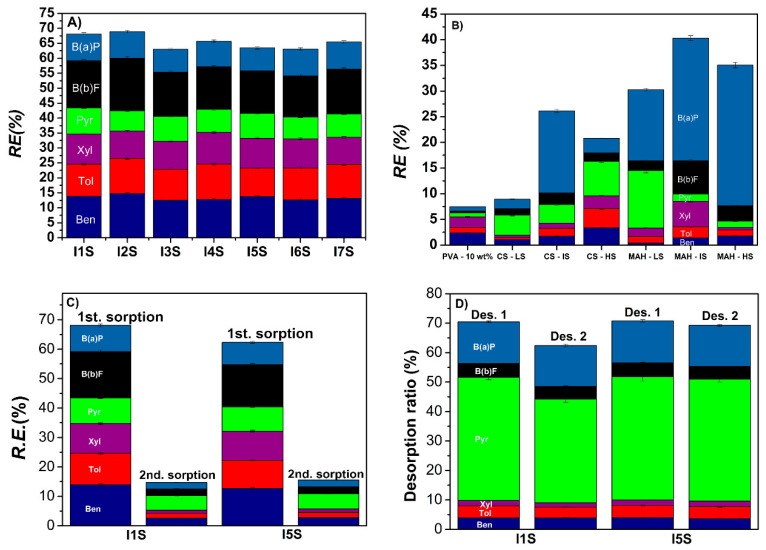
(**A**) Dependence on the adsorption capacity of the PVA/CS/MA-β-CD hydrogel with the intermediate swelling degree of solutions with the different initial concentration of BTXs and PAHs (see Table S1), at 25 °C; (**B**) PVA 10 wt % and blanks compositions, see the text for the meaning of blanks; (**C**) comparative results on the removal efficiency (RE) of the BTXs and PHAs by the hydrogels based on PVA/CS/MA-β-CD (IS), (I1S and I5S), after the first desorption (Des.1); and (**D**) desorption results for six miscible organics (BTXs and PAHs on PVA/CS/MA-β-CD hydrogels (IS).

The removal efficiency (*RE*) of BTXs and PAHs by PVA/CS/MA-β-CD hydrogel with an intermediate swelling degree was also evaluated (Figure 8A). The removal efficiency was higher than 60% for all initial concentration of BTXs and PAHs. It is also worth noting that the experiments performed with the hydrogels of a lower and higher swelling degree presented variable results (e.g., cumulative *RE* to LS and HS hydrogel are 43% and 19%, respectively; see Figure S5). These hydrogels revealed improved capacity to removal petroleum pollutant compounds in comparison to hydrogels containing pectin/chitosan [33].

The removal efficiency of these adsorbates was evaluated for blanks containing only PVA 10 wt % and reference hydrogels (blanks; Table S1 and Figure 8B). It is interesting to mention that these materials removed a maximum of 40% of the initial amount of PAHs and BTX samples. Including, PVA 10% was the sample with a lower removal capacity. These results reinforce the importance of the hydrogels of PVA/CS/MA-β-CD on the aromatics pollutants removal capacity. Likewise, these results agree with swelling studies (see Section 3.2 and Section 3.3.1), which showed the strong influence of all polymers on the swelling.

Table 4 shows the performance of different adsorbents for adsorption of PAHs and BTXs. Despite the importance of the work previously reported on this issue, it is worth noticing that the compassion with the data reported by us was limited once data summarized in the Table 4 did not regard a simultaneous sorption of different adsorbates.

#### 3.4.3. Sorption–Desorption Cycles

The desorption process is an important aspect that allows the evaluation of the reuse of the sorbent and may contribute to a well understanding of the sorption mechanism [8]. Figure 8C shows that the 2nd sorption process was relatively much lower for all aromatic hydrocarbons when compared to the 1st sorption. On the other hand, from the analysis of desorption ratio (Figure 8D), PAHs were significantly desorbed from hydrogels, with *DR* higher than 62%, being pyrene the PAHs with the highest desorption rate (41%). However, BTXs were meaningfully retained by the hydrogels; this same fact was also observed in previous sorption studies of BTXs and PAHs using the aerogel-MTMS and the hydrogel based on pectin/chitosan [8,33]. The analysis of these data also suggests that the BTXs, maybe because of the presence of two amphiphilic compounds (β-CD [33] and PVA [27]) or due to steric reasons, could contribute to a better dissolution of BTXs at the hydrogel interface and thus these compounds were also less able to be desorbed. Although desorption results suggest that the reuse of hydrogels was not possible, the removal of sorbed hydrocarbon seemed to be effective.

### 3.5. Performance of Hydrogels Towards Water Contaminated with A Real Petroleum Sample

Here we intended to evaluate the performance of the PVA/CS/MA-β-CD (IS) composite hydrogel for the removal of petroleum derivative compounds (BTXs and PAHs) from a commercial gasoline sample. Table 5 presents the initial concentrations (*C*_0_) of those compounds in the sample. 

Analysis of simultaneous sorption demonstrates that the removal efficiency (RE) for PAHs was much higher than that observed for BTXs (cumulative sorbed concentration of BTXs and PAHs were, respectively, ca. 1.48% and ca. 25.11%). Thus, the low BTXs RE might have been influenced by the presence of a considerable number of monoaromatic compounds in commercial gasoline [87]. 

## 4. Conclusions

An amphiphilic coacervate physically crosslinked hydrogel of PVA/CS/MA-β-CD was synthesized. The amphiphilic features were provided using the β-cyclodextrin and PVA. For that the β-CD was initially functionalized (28% yield) with maleic acid. Furthermore, from the FTIR analysis it could be concluded that the structure of β-CD remained unchanged upon functionalization.

The obtained hydrogels show a significant dependence of the swelling on the different polymers. A multivariate analysis showed that the increase in the swelling was due to the occurrence of synergistic effects and variables (MA-β-CD and CS). The swelling kinetics was evaluated and could be well described by a pseudo-first-order kinetic model, revealing that water–water interactions were stronger than water–polymer interactions, which may be justified by the effect of PVA on the hydrogel.

It was found that the MA-β-CD was directly related to the presence of randomly interconnected pores (ranging from 5.7 to 36.1 μm) in the surface of the hydrogels. Rheological characterization showed that mechanical strength was higher for the hydrogel with an intermediate swelling degree. Additionally, the greater amount of CS was crucial for the hydrogel stiffness. *G*’ and *G*’’ evaluation indicates that the hydrogels were stable in the temperature range from 20 to 60 °C. 

The hydrogel with an intermediate swelling degree was used to investigate the simultaneous sorption isotherms and kinetics of six different petroleum-based contaminants (PHAs and BTXs). The sorption kinetics follows a pseudo-1^st^ order kinetics suggesting that the solvent played a major role on the hydrogel matrix. This is in close agreement with the observed Henry-based isotherms, showing that the interaction between adsorbent–adsorbate was weak compared to the polymer–polymer interaction.

The cumulative removal efficiency for the compounds BTXs and HPAs was 43%, 69%, and 19% for low, intermediate, and high swelling hydrogels, respectively. For the hydrogel with the best removal percentage, benzene, toluene, and B(b)F were the compounds with high partial removal efficiencies: 13.4%, 11%, and 15%, respectively.

PAHs were significantly desorbed from hydrogels (desorption higher than 62%); from all adsorbates, BTXs were those mostly retained. This can be justified by the amphiphilic characteristics of β-CD and PVA, which, concomitantly to steric reasons, promoted better interactions with BTXs.

The removal of BTXs and PAHs from a commercial gasoline sample was shown as promising, despite the high complexity of the sample, and is encouraging for the application of these hydrogels for the application in environmental conditions.

## Figures and Tables

**Table 1:** Concentration of the different adsorbates in the solutions used for sorption kinetics experiments^**^.

*C_0_* (mg L^−1^)						
	Benzene	Xylenes	Toluene	Pyrene	B(b)F	B(a)P
**IS** *						
I1K	6.9 (±0.2) *	8.1 (±0.4)	8.4 (±0.3)	2.2 (±0.1)	2.4 (±0.1)	2.2 (±0.1)
I2K	9.9 (±0.3)	14.4 (±0.4)	10.2 (±0.3)	4.0 (±0.1)	4.3 (±0.1)	4.7 (±0.1)
I3K	12.7 (±0.4)	15.9 (±0.5)	12.5 (±0.4)	6.3 (±0.1)	6.2 (±0.1)	5.9 (±0.1)
I4K	32 (±1)	33.4 (±0.9)	32 (±1)	10.1 (±0.3)	9.31 (±0.36)	9.9 (±0.2)
I5K	48.1 (±1.4)	50.1 (±1.5)	48.0 (±1.2)	15.1 (±0.5)	13.9 (±0.4)	13.0 (±0.3)

* values inside brackets are standard deviations of the average. ** PVA/CS/MA-β-CD hydrogel showing the intermediate swelling degree (IS).

**Table 2:** Kinetic parameters for the swelling of water by PVA/CS/MA-β-CD-based blends gels, at 25 °C (see Table S2).

		Equation (6)			Equation (7)		
Form	*Q_e,exp_*	*Q_e,1_*	*k_1,w_* (10^−4^s^−1^)	*AIC*	*Q_e,2_*	*k_2,w_* (10^−5^ s^−1^)	*AIC*
1	1.91 (±0.04)	1.3 (±0.1)	2.1 (±0.1)	4.46	2.10 (±0.04)	0.02 (±0.01)	9.48
2	7.2 (±0.1)	4.9 (±0.1)	2.2 (±0.1)	4.44	7.92 (±0.01)	0.003 (±0.002)	8.30
3	0.14 (±0.04)	0.1 (±0.1)	2.2 (±0.1)	4.51	0.16 (±0.04)	1.23 (±0.02)	11.79
4	1.98 (±0.03)	1.4 (±0.1)	2.2 (±0.1)	4.46	2.2 (±0.1)	0.02 (±0.06)	10.12
5	3.7 (±0.1)	2.6 (±0.1)	2.3 (±0.1)	4.38	4.07 (±0.02)	0.08 (±0.03)	9.41
6	2.7 (±0.1)	2.0 (±0.1)	2.3 (±0.1)	4.46	2.94 (±0.03)	0.04 (±0.01)	9.45
7	2.2 (±0.1)	1.6 (±0.1)	2.3 (±0.1)	4.36	2.41 (±0.03)	0.029 (±0.003)	9.32
8	23.5 (±0.4)	16.8 (±0.1)	2.2 (±0.1)	4.44	25.73 (±0.03)	0.0003 (±0.0001)	7.26
9	2.4 (±0.1)	1.8 (±0.1)	2.3 (±0.1)	4.37	2.69 (±0.03)	0.04 (±0.03)	9.57
PVA	4.08 (±0.12)	2.7 (±0.1)	3.09 (±0.04)	3.66	4.37 (±0.04)	0.002 (± 0.002)	9.91

**Table 3:** Freundlich and Henry parameters obtained by fitting Equations (8) and (9) to the experimental data ^*^ (see Figure S4).

		Freundlich				Henry		
IS **	*C*_0_ (mg L^−1^)	*1/n_F_*	*K_F_* (mg ^(n−1)/n^ L^1/n^ g^−1^)	*R* ^2^	*AIC*	*K_H_* (L g^−1^)	R^2^	*AIC*
Benzene	6.9–52.8	1.04 (±0.09) *	0.07 (±0.02)	0.9308	4.04	0.09 (±0.003)	0.9924	3.39
Xylenes	13.1–55.1	1.006 (±0.004)	0.026 (±0.004)	0.9830	5.16	0.027 (±0.001)	0.9858	3.76
Toluene	8.4–52.4	0.97 (±0.05)	0.032 (±0.005)	0.9731	3.63	0.028 (±0.001)	0.9862	2.77
B(b)F	2.4–15.4	1.21 (±0.07)	0.027 (±0.004)	0.9594	3.56	0.038 (±0.002)	0.9890	2.98
Pyrene	3.4–16.6	0.98 (±0.06)	0.022 (±0.003)	0.9638	3.47	0.021 (±0.001)	0.9696	2.38
B(a)P	1.9–7.3	1.12 (±0.02)	6.94 (±0.2) × 10^−3^	0.9984	3.49	0.023 (±0.001)	0.9784	2.46

* values inside brackets are standard deviations of the average. ** PVA/CS/MA-β-CD hydrogel that presented intermediate (IS) swelling degree.

**Table 4:** Summary of removal efficiencies of hydrocarbons by different adsorbents.

Hydrocarbon	Adsorbent	Removal Efficiency (%)	
**Benzene**	cupric oxide nanoparticles	98.7	[75]
	activated carbon	98.89	[76]
	zeolite	35	[77]
**Toluene**	cupric oxide nanoparticles	92.5	[75]
	activated carbon	99.86	[76]
	zeolite	55	[77]
**Xylenes**	activated carbon	99.99	[76]
	zeolite	77 */99 **	[77]
	crumb rubber	81	[78]
**Pyrene**	modified pine bark	55.28–93.53	[79]
	mineral surfaces	25	[80]
	Brazilian peat	75.5–92.1	[81]
**B(a)P**	hardwood mulch	92	[82]
	mineral surfaces	82	[80]
	Porous organoclay composite	99	[83]
**B(b)F**	activated carbon	72	[84]
	wood ashes	98.6	[85]
	mesoporous materials	32	[86]

* o-xylene; ** p-xylene.

**Table 5:** Adsorption parameters of aromatic hydrocarbons in a fossil fuel sample by PVA/CS/MA-β-CD hydrogel with intermediate swelling degree.

	C_0_ in the Used Fossil Fuel Before Dilution (wt-%)^43^	C_0_ in the Spiked Solution (mg L^−1^)	Removal Efficiency (%)	Q_e,exp_ (10^−2^ mg g^−1^)
Benzene	0.08 (±0.01) *	4.1 (±0.1)	0.37 (±0.01)	0.4 (±0.1)
Xylenes	1.32 (±0.04)	11.2 (±0.4)	0.28 (±0.01)	0.95 (±0.02)
Toluene	0.78 (±0.01)	7.4 (±0.2)	0.83 (±0.02)	1.71(±0.03)
Pyrene	0.012 (±0.001)	1.3 (±0.1)	4.3 (±0.1)	0.48 (±0.01)
B(a)P	5.0 (±0.2) × 10^−3^	0.49 (±0.02)	9.8 (±0.2)	5.8 (±0.1)
B(b)F	4.0 (±0.1) × 10^−4^	0.31 (±0.03)	11.02 (±0.22)	0.3 (±0.1)

* values inside brackets are standard deviations of the average.

## Supplementary Materials

The following are available online at https://www.mdpi.com/2073-4360/12/4/877/s1, Figure S1: (A) Blends of CS, PVA and MA-β-CD in wells of cell culture plates before freeze-thawing cycles. (B) Composite hydrogels based on CS/PVA/MA-β-CD swollen in ultrapure water and after drying, respectively, Figure S2: ATR-FTIR spectra of β-CD, MA and MA-β-CD (I, II and III), respectively, Figure S3: Graphs of the fitting of linearized forms of pseudo-first (■) and pseudo-second (□) order equations to experimental sorption data for BTXs ((A) Ben (B), Tol (C) and Xyl (D)) and PAHs (Pyr (E), (B(b)F and (F) B(a)P) onto the PVA/CS/MA-β-CD hydrogel with intermediate swelling degree, at 25 °C, Figure S4: Sorption isotherms of BTXs and some PAHs, from aqueous solutions, by the PVA/CS/MA-β-CD hydrogels with low (A), intermediate (B) and high (C) swelling degree, at 25 °C. Red dashed lines and black solid correspond to the fitting of Henry and Freundlich models to the experimental data, respectively. (A) to (C): Ben (■), Xyl (▲), Tol (●), Pyr (☐), B(a)P (△) and B(b)F (o), Figure S5: Adsorption capacity dependence of the PVA/CS/MA-β-CD hydrogels with low (A) and high (B) swelling degree on the initial concentration of benzene, toluene, xylenes and some PAHs, at 25 °C, Table S1: Composition of the BTXs and PHAs solutions mixed used to obtain the sorption isotherms at 25 °C, Table S2: Formulation of composite hydrogels, Table S3: Analysis of variance using the Design Expert© software related to the dependent variables, at the confidence level of 95 %, for the response surface of the star planning with two factors, for swelling degree (Q), Table S5: Freundlich and Henry parameters obtained by fitting Equations (7) and (8) to the experimental data (Figure S2).
