# Supplementary material for: Uncommon Sorption Mechanism of Aromatic Compounds onto Poly(Vinyl Alcohol)/Chitosan/Maleic Anhydride-β-Cyclodextrin Hydrogels
Source: Polymers (Basel). 2020 Apr 10;12(4):877. doi: 10.3390/polym12040877 (PMC7652220; doi:10.3390/polym12040877)
Supplement: Supplementary file 1 [file polymers-12-00877-s001.pdf]

Supplementary Materials (SI)†

Uncommon sorption mechanism of aromatic compounds onto poly(vinyl alcohol)/chitosan/ maleic anhydride-β-cyclodextrin hydrogels

Cesar M. C. Filho [1,2*], Pedro V. A. Bueno [3], Alan F. Y. Matsushita [1], Bruno H. Vilsinski [1,3] Adley F. Rubira [3], Edvani C. Muniz [3,4], Dina M. B. Murtinho [1] and Artur J.M.Valente [1,*]

[1]*CQC, Department of Chemistry, 3004-535, Coimbra, Portugal.*

[2]*BRinova Biochemistry Lda.,7005- 485 Évora, Portugal.*

[3]*Grupo de Materiais Poliméricos e Compósitos (GMPC) - Departamento de Química, Universidade Estadual de Maringá, UEM, 87020-900, Maringá, PR, Brazil.*

[4]*Post-graduate Program on Materials Science & Engineering, Federal University of Technology, Paraná (UTFPR-LD), 86036-370, Londrina, PR, Brazil.*

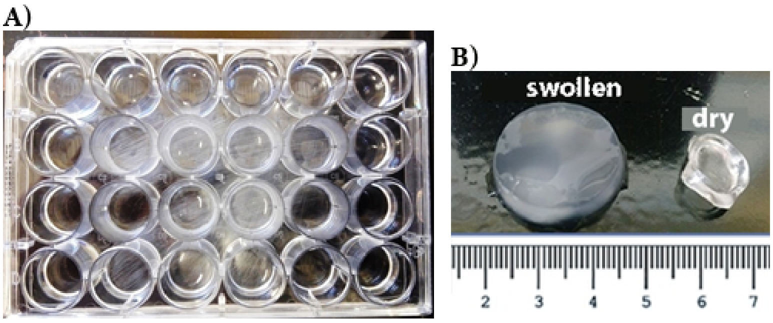

Figure S1: (A) Blends of CS, PVA and MA-β-CD in wells of cell culture plates before freeze-thawing cycles. (B) Composite hydrogels based on CS/PVA/MA-β-CD swollen in ultrapure water and after drying, respectively.

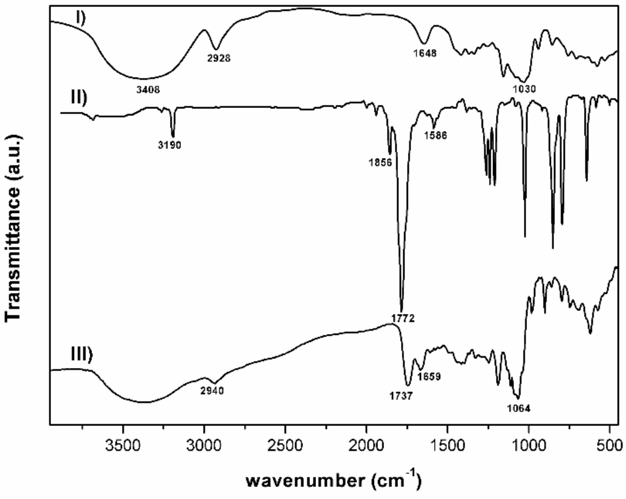

Figure S2: ATR-FTIR spectra of β-CD, MA and MA-β-CD (I, II and III), respectively.

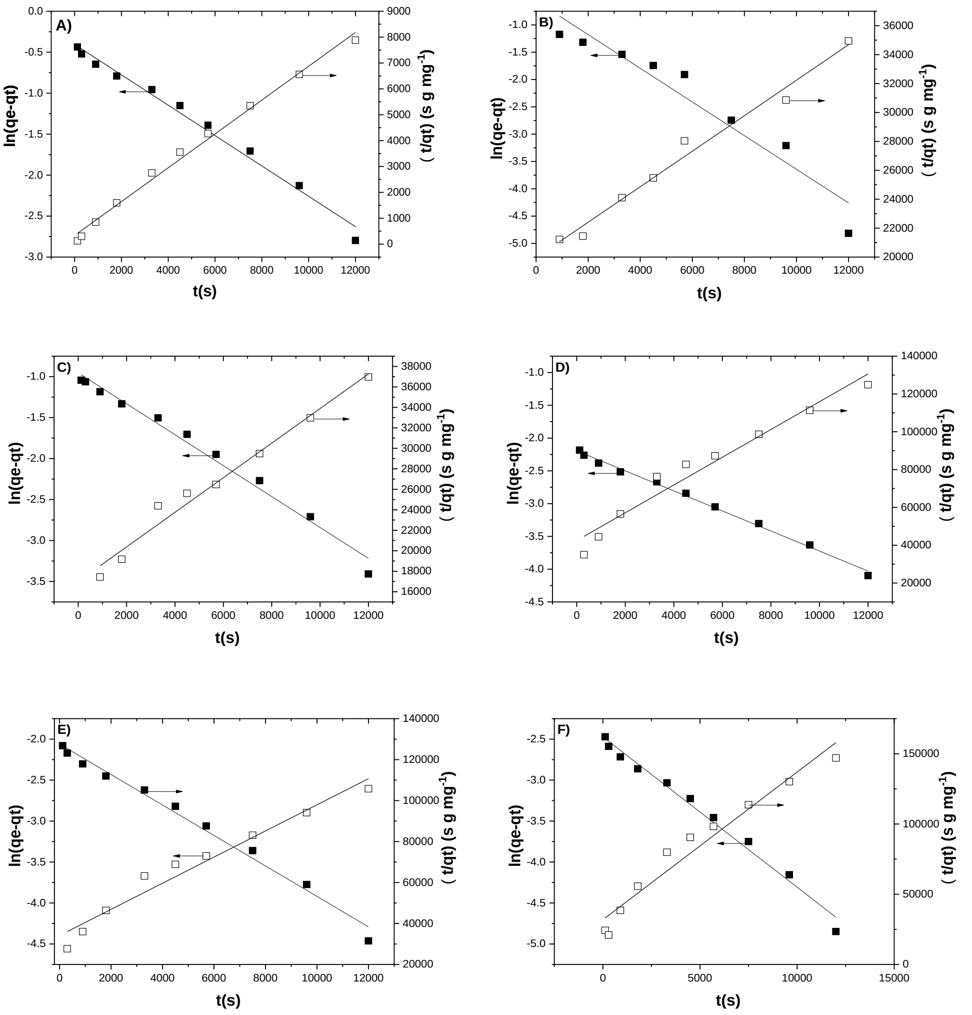

Figure S3: Graphs of the fitting of linearized forms of pseudo-first (■) and pseudo-second (□) order equations to experimental sorption data for BTXs ((A) Ben (B), Tol (C) and Xyl (D)) and PAHs (Pyr (E), (B(b)F and (F) B(a)P) onto the PVA/CS/MA-β-CD hydrogel with intermediate swelling degree, at 25 ºC.

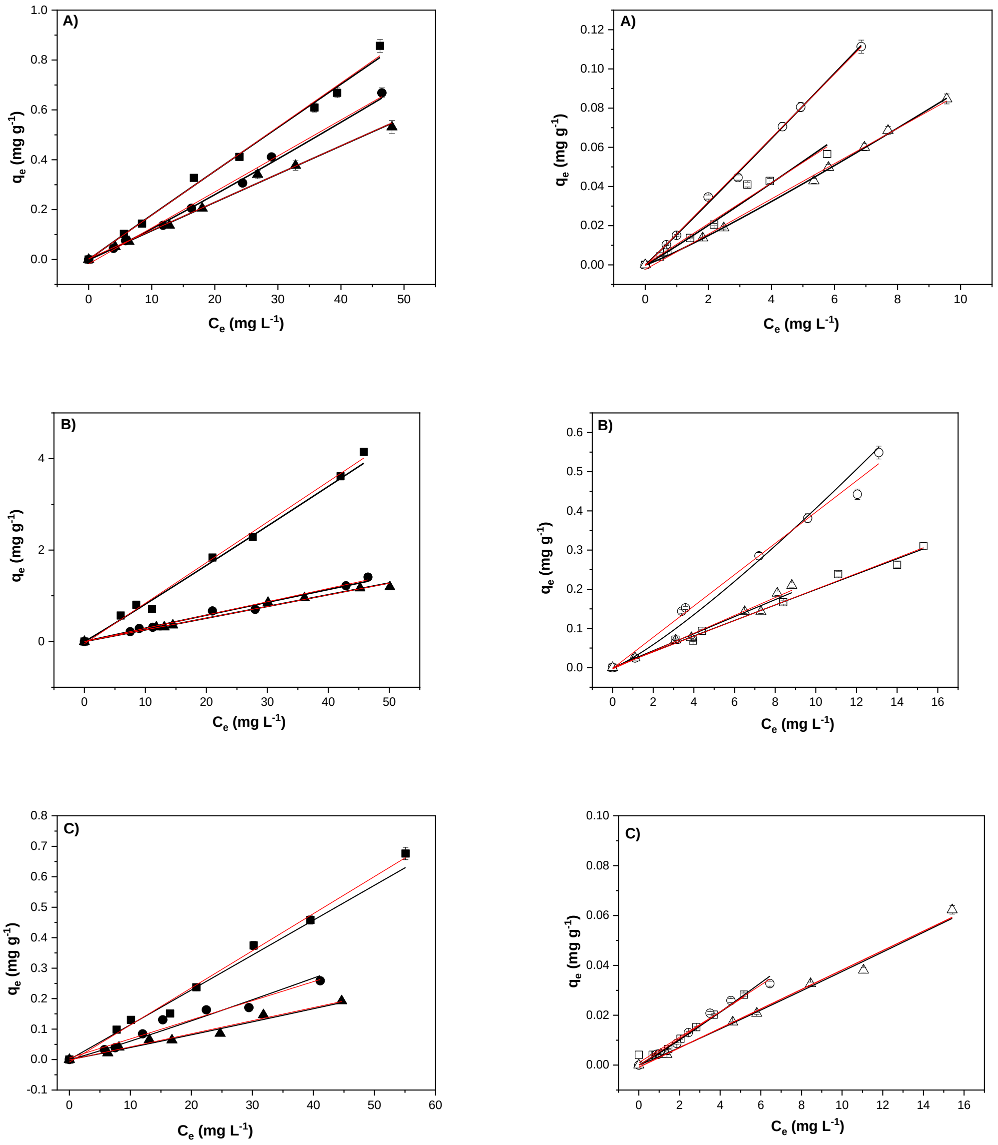

Figure S4: Sorption isotherms of BTXs and some PAHs, from aqueous solutions, by the PVA/CS/MA-β-CD hydrogels with low (A), intermediate (B) and high (C) swelling degree, at 25ºC. Red dashed lines and black solid correspond to the fitting of Henry and Freundlich models to the experimental data, respectively. (A) to (C): Ben (■),Xyl (▲), Tol (●), Pyr (☐), B(a)P (△) and B(b)F (o).

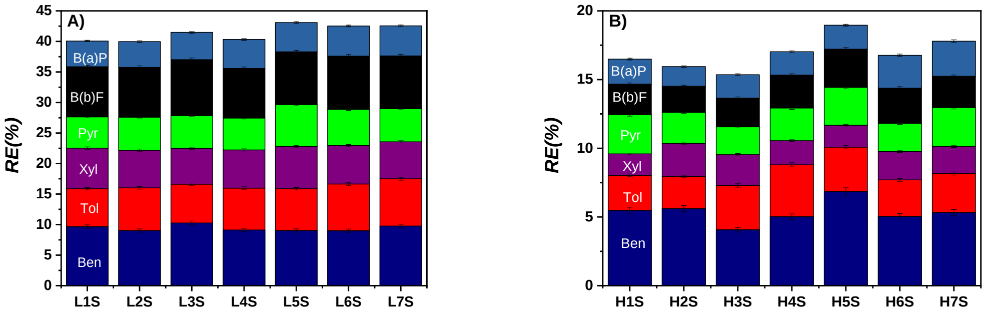

Figure S5: Adsorption capacity dependence of the PVA/CS/MA-β-CD hydrogels with low (A) and high (B) swelling degree on the initial concentration of benzene, toluene, xylenes and some PAHs, at 25 ºC.

Table S1: Composition of the BTXs and PHAs solutions mixed used to obtain the sorption isotherms at 25 ºC.

Composite hydrogels	C_0 (mg L^{-1})					
**LS****	Benzene	Xylenes	Toluene	Pyrene	B(a)P	B(b)F
L1S	6.2 (±0.2)	4.5 (±0.1)	4.2 (±0.1)	0.49 (±0.01)	1.9 (±0.1)	0.73 (±0.02)
L2S	9.3 (±0.2)	6.8 (±0.2)	6.3 (±0.2)	0.74 (±0.02)	2.6 (±0.1)	1.08 (±0.03)
L3S	18.6 (±0.4)	13.6 (±0.3)	12.6 (±0.3)	1.5 (±0.3)	5.6 (±0.1)	2.2 (±0.1)
L4S	26.3 (±0.4)	19.2 (±0.4)	17.5 (±0.4)	2.3 (±0.1)	6.1 (±0.2)	3.2 (±0.2)
L5S	39.4 (±0.7)	29 (±1)	26.2 (±0.5)	3.5 (±0.1)	7.3 (±0.2)	4.6 (±0.1)
L6S	43.3 (±1.3)	35 (±1)	31.4 (±0.9)	4.2 (±0.1)	8.1 (±0.2)	5.4 (±0.2)
L7S	51.2 (±2.6)	51.2 (±1)	50.4 (±1.5)	6.1 (±0.3)	10.05 (±0.20)	7.5 (±0.2)
**IS****						
I1S	6.9 (±0.3)	13.1 (±0.7)	8.4 (±0.4)	3.4 (±0.2)	1.2 (±0.1)	2.4 (±0.1)
I2S	10.0 (±0.3)	14.4 (±0.4)	10.2 (±0.3)	4.2 (±0.1)	3.4 (±0.1)	4.2 (±0.1)
I3S	12.7 (±0.4)	15.9 (±0.5)	12.5 (±0.4)	4.8 (±0.1)	4.2 (±0.1)	4.4 (±0.1)
I4S	24.1 (±1.2)	33.7 (±1.5)	23.8 (±1.2)	9.1 (±0.5)	7.1 (±0.2)	8.4 (±0.4)
I5S	32.03 (±0.96)	40.1 (±0.2)	30.9 (±1.2)	12.1 (±0.3)	7.9 (±0.4)	11.2 (±0.3)
I6S	48.1 (±1.2)	50.1 (±1.5)	48 (±1)	15.1 (±0.4)	8.9 (±0.3)	13.9 (±0.4)
I7S	52.8 (±1.1)	55.1 (±1.2)	52.4 (±1.1)	16.6 (±0.3)	9.7 (±0.2)	15.4 (±0.3)
**HS****						
H1S	8.2 (±0.3)	6.4 (±0.2)	5.9(±0.2)	0.70 (±0.02)	1.1 (±0.1)	0.90 (±0.03)
H2S	10.7 (±0.5)	8.3 (±0.4)	7.7(±0.2)	0.84 (±0.04)	1.4 (±0.1)	1.0 (±0.1)
H3S	17.2 (±0.3)	13.4 (±0.3)	12.4(±0.2)	1.48 (±0.03)	4.7 (±0.2)	1.91 (±0.04)
H4S	21.9 (±0.2)	17.1 (±0.4)	15.9(±0.4)	2.1 (±0.1)	5.9 (±0.2)	2.5 (±0.1)
H5S	32.1 (±0.9)	25.1 (±0.8)	23.2(±0.7)	2.9 (±0.2)	8.6 (±0.1)	3.6 (±0.2)
H6S	41.6 (±0.8)	32.5 (±0.7)	30.2(±0.6)	3.8 (±0.2)	11.2 (±0.2)	4.7 (±0.2)
H7S	58.2 (±1.2)	45.5 (±0.9)	42.3(±0.8)	5.3 (±0.2)	15.7 (±0.3)	6.6 (±0.2)
Blanks						
PVA (10 *wt%*)	33.52(±1.01)	34.35(±1.03)	30.9(±0.6)	4.9(±0.2)	7.3(±0.2)	4.3(±0.1)
PVA/CS LS	42.6(±1.3)	28.8(±0.9)	26.5(±0.8)	3.1(±0.1)	7.9(±0.2)	4.6(±0.1)
PVA/CS IS	29.1(±0.9)	26.5(±0.8)	26.3(±0.5)	4.6(±0.1)	5.5(±0.2)	4.8(±0.1)
PVA/CS LS	42.6(±1.3)	32.7(±0.9)	29.8(±0.6)	3.9(±0.1)	7.8(±0.2)	4.8(±0.1)
PVA/MA-β-CD LS	33.1(±0.9)	34.19(±1.03)	31.2(±0.6)	5.1(±0.2)	6.9(±0.2)	4.5(±0.1)
PVA/MA-β-CD IS	6.4(±0.2)	13.5(±0.4)	9.6(±0.2)	3.8(±0.1)	1.18(±0.04)	4.1(±0.1)
PVA/MA-β-CD HS	16.3(±0.5)	25.3(±0.8)	17.8(±0.4)	6.9(±0.2)	3.1(±0.1)	5.5(±0.2)

*values inside brackets are standard deviations of the average.
**PVA/CS/MA-β-CD hydrogel that presented lower (LS), intermediate (IS) and high swelling degree (HS).

Table S2: Formulation of composite hydrogels.

**Form.**	**CS (% *v/v*)**	**MA-β-CD (% *w/v*)**	**PVA (% *v/v*)**
1	0.05 (-1.41)*	5.75 (0)	94.2
2	1.70 (-1)	1.70 (-1)	96.6
3	1.70 (-1)	9.75 (+1)	88.55
4	5.75 (0)	11.40 (+1.41)	82.85
5	5.75 (0)	0.05 (-1.41)	94.2
6	5.75 (0)	5.75 (0)	88.5
7	9.75 (+1)	1.70 (-1)	88.55
8	9.75 (+1)	9.75 (+1)	80.5
9	11.40 (+1.41)	5.75 (0)	82.85
PVA	0	0	100

*Values inside brackets are coded levels of the factorial study.

The analysis of the significance of the effects of the independent variables (MA-β-CD and CS) on swelling degree was evaluated through the Design Expert© software (trial version) to carry out the analysis of the variance (ANOVA) of the obtained results, as well as to perform the graphic optimization procedures. In this context, the *F*-test was used as a criterion for validating the statistical significance of the models obtained at the 95 % confidence level. Statistical analysis was also used for estimation of the experimental errors (Table S3).

Table S3: Analysis of variance using the Design Expert© software related to the dependent variables, at the confidence level of 95 %, for the response surface of the star planning with two factors, for swelling degree (Q).

**Variation Source**	**Sum of squares**	**Mean square**	**F value**	***p-value (Prob>F)***
Model	226.48	75.49	2.44	0.1619
A*	79.78	79.78	2.58	0.1592
B	39.83	39.83	1.29	0.2995
AB	175.39	175.39	5.68	0.0454
Residual	185.32	30.89		
Total	411.80			

*A: CS; B: MA-β-CD.

Table S4: Fitting kinetic parameters for the simultaneous sorption of mono- and polycyclic aromatic hydrocarbons onto the composite hydrogels based on PVA/CS/MA-β-CD with intermediate swelling degree, at 25 ºC.

	$q_{e,exp}$ (mg g^{-1})	$q_{e,1}$ (mg g^{-1})	k_1 (10^{-4} g mg^{-1} s^{-1})	*AIC*	$q_{e,2}$ (mg g^{-1})	k_2 (10^{-4} g mg^{-1} s^{-1})	*AIC*
**Benzene**							
I1K	0.160 (±0.003)*	0.07 (±0.04)	1.85 (±0.07)	4.71	0.16 (±0.04)	0.32 (±0.02)	12.77
I2K	0.29 (±0.01)	0.12 (±0.04)	1.84 (±0.05)	4.79	0.30 (±0.02)	1.21 (±0.06)	12.16
I3K	0.26 (±0.01)	0.15 (±0.02)	2.72 (±0.21)	4.84	0.26 (±0.02)	0.89 (±0.04)	12.88
I4K	1.58 (±0.03)	0.67 (±0.04)	1.85 (±0.07)	4.79	1.59 (±0.01)	35.2 (±0.1)	10.70
I5K	2.53 (±0.05)	1.05 (±0.02)	1.85 (±0.06)	3.79	2.49 (±0.04)	381 (±1)	9.25
**Xylenes**							
	$q_{e,exp}$ (mg g^{-1})	$q_{e,1}$ (mg g^{-1})	k_1 (10^{-4} g mg^{-1} s^{-1})	*AIC*	$q_{e,2}$ (mg g^{-1})	k_2 (10^{-4} g mg^{-1} s^{-1})	*AIC*
I1K	0.17 (±0.03)	0.18 (±0.04)	1.9 (±0.1)	3.89	0.18 (±0.11)	0.07 (±0.02)	14.32
I2K	0.35 (±0.01)	0.38 (±0.04)	1.9 (±0.1)	3.90	0.38 (±0.12)	0.24 (±0.02)	13.76
I3K	0.4 (±0.1)	0.39 (±0.04)	1.9 (±0.1)	3.91	0.4 (±0.1)	0.27 (±0.01)	13.44
I4K	1.27 (±0.04)	1.4 (±0.1)	1.82 (±0.01)	4.17	1.38 (±0.02)	3.12 (±0.04)	12.79
**Toluene**							
	$q_{e,exp}$ (mg g^{-1})	$q_{e,1}$ (mg g^{-1})	k_1 (10^{-4} g mg^{-1} s^{-1})	*AIC*	$q_{e,2}$ (mg g^{-1})	k_2 (10^{-3} g mg^{-1} s^{-1})	*AIC*
I1K	0.22 (±0.04)	0.28 (±0.02)	2.62 (±0.03)	5.10	0.23 (±0.01)	0.012 (±0.011)	13.97
I2K	0.35 (±0.01)	0.45 (±0.02)	2.79 (±0.03)	5.0	0.4 (±0.1)	0.034 (±0.002)	13.40
I3K	0.46 (±0.04)	0.65 (±0.02)	3.01 (±0.04)	5.17	0.5 (±0.1)	0.05 (±0.04)	13.40
I4K	2.06 (±0.04)	3.02 (±0.02)	2.98 (±0.03)	5.19	2.2 (±0.1)	0.84 (±0.04)	12.66
I5K	2.9 (±0.1)	2.93 (±0.03)	4.38 (±0.02)	5.22	3.16 (±0.02)	1.73 (±0.02)	12.45
**Pyrene**							
	$q_{e,exp}$ (mg g^{-1})	$q_{e,1}$ (mg g^{-1})	k_1 (10^{-4} g mg^{-1} s^{-1})	*AIC*	$q_{e,2}$ (mg g^{-1})	k_2 (10^{-5} g mg^{-1} s^{-1})	*AIC*
I1K	0.04 (±0.01)	0.04 (±0.02)	1.54 (±0.03)	3.22	0.04 (±0.01)	0.04 (±0.01)	14.67
I2K	0.08 (±0.03)	0.08 (±0.02)	1.54 (±0.02)	3.24	0.08 (±0.02)	0.20 (±0.01)	14.05
I3K	0.11 (±0.02)	0.11 (±0.02)	1.53 (±0.03)	3.16	0.12 (±0.01)	0.32 (±0.02)	13.61
I4K	0.35 (±0.01)	0.33 (±0.02)	1.54 (±0.04)	3.24	0.36 (±0.04)	3.64 (±0.01)	12.77

I5K	0.51 (±0.02)	0.49 (±0.02)	1.54 (±0.03)	3.22	0.54 (±0.02)	7.02 (±0.02)	12.38
			B(a)P				
	$q_{e,exp}$ (mg g^{-1})	$q_{e,1}$ (mg g^{-1})	k_1 (10^{-4} g mg^{-1} s^{-1})	*AIC*	$q_{e,2}$ (mg g^{-1})	k_2 (10^{-5} g mg^{-1} s^{-1})	*AIC*
I1K	0.03 (±0.01)	0.03 (±0.04)	1.9 (±0.1)	3.85	0.03 (±0.01)	0.04 (±0.02)	14.81
I2K	0.08 (±0.04)	0.07 (±0.04)	1.9 (±0.1)	3.84	0.08 (±0.01)	0.26 (±0.01)	14.01
I3K	0.09 (±0.01)	0.09 (±0.04)	1.9 (±0.1)	3.83	0.09 (±0.01)	0.30 (±0.02)	13.87
I4K	0.28 (±0.01)	0.26 (±0.04)	1.86 (±0.04)	3.85	0.29 (±0.04)	3.34 (±0.04)	12.91
I5K	0.41 (±0.02)	0.35 (±0.04)	1.9 (±0.1)	3.96	0.42 (±0.03)	8.72 (±0.03)	12.56
			B(b)F				
	$q_{e,exp}$ (mg g^{-1})	$q_{e,1}$ (mg g^{-1})	k_1 (10^{-4} g mg^{-1} s^{-1})	*AIC*	$q_{e,2}$ (mg g^{-1})	k_2 (10^{-4} g mg^{-1} s^{-1})	*AIC*
I1K	0.05 (±0.01)	0.05 (±0.02)	1.86 (±0.04)	3.79	0.05 (±0.03)	0.006 (±0.003)	14.16
I2K	0.095 (±0.003)	0.094 (±0.003)	1.9 (±0.1)	3.79	0.1 (±0.2)	0.03 (±0.01)	13.98
I3K	0.125 (±0.003)	0.13 (±0.04)	1.9 (±0.1)	3.79	0.14 (±0.01)	0.05 (±0.01)	13.59
I4K	0.36 (±0.01)	0.35 (±0.02)	1.9 (±0.1)	3.79	0.37 (±0.03)	0.46 (±0.02)	12.67
I5K	0.53 (±0.02)	0.52 (±0.04)	1.9 (±0.1)	3.79	0.55 (±0.02)	1.00 (±0.01)	12.31

Table S5: Freundlich and Henry parameters obtained by fitting Eqs. (7) and (8) to the experimental data (Figure S2).

		**Freundlich**			**Henry**	
**LS****	C_0 (mg L^{-1})	$1/n_F$	K_F (mg$^{(n-1)/n}$ L$^{1/n}$ g^{-1})	R^2	K_H (L g^{-1})	R^2
Benzene	6.2-51.2	0.98(±0.03)	0.019(±0.002)*	0.9921	0.012(± 0.004)	0.9969
Xylenes	4.5-51.2	0.98(±0.03)	0.012(±0.001)	0.9915	0.012(± 0.003)	0.9924
Toluene	4.2-50.4	1.07(±0.03)	0.011(±0.001)	0.9926	0.015(± 0.004)	0.9938
Pyrene	0.5-6.1	1.04(±0.04)	9.8(±0.4) $\times 10^{-3}$	0.9817	0.0105(± 0.0006)	0.9718
B(a)P	1.9-7.3	1.12(±0.02)	6.92(±0.2) $\times 10^{-3}$	0.9985	0.0091(± 0.0002)	0.9961
B(b)F	0.7-5.4	1.04(±0.02)	1.57(±0.04) $\times 10^{-2}$	0.9956	0.017(± 0.003)	0.9984
**HS****	C_0 (mg L^{-1})	$1/n_F$	K_F (mg$^{(n-1)/n}$ L$^{1/n}$ g^{-1})	R^2	K_H (L g^{-1})	R^2
Benzene	8.2-58.2	1.002(±0.08)	0.011(±0.003)	0.9353	0.013(±0.001)	0.9905
Xylenes	6.4-45.5	1.03(±0.09)	0.004(±0.001)	0.9253	0.0044(±0.0003)	0.9708
Toluene	5.9-42.3	1.08(±0.09)	0.005(±0.001)	0.9319	0.007(±0.001)	0.9595
Pyrene	0.7-5.3	1.02(±0.06)	0.0051(±0.0003)	0.9681	0.0052(±0.0004)	0.9653
B(a)P	1.1-15.7	1.05(±0.04)	0.0034(±0.0002)	0.9798	0.0038(±0.0002)	0.9891
B(b)F	0.9-6.6	1.09(±0.05)	0.0047(±0.0002)	0.9818	0.0055(±0.0003)	0.9844

*values inside brackets are standard deviations of the average.

**PVA/CS/MA-β-CD hydrogel that presented lower (LS) and high swelling degree (HS).
